# Craniomaxillofacial morphology alterations in children, adolescents and adults with neurofibromatosis 1: A cone beam computed tomography analysis of a Brazilian sample

**DOI:** 10.4317/medoral.22155

**Published:** 2018-02-25

**Authors:** Eloá-Borges Luna, Maria-Elisa-Rangel Janini, Flávia Lima, Raquel-Richelieu-de Andrade Pontes, Fábio-Ribeiro Guedes, Mauro Geller, Licínio-Esmeraldo da Silva, Alexandre-Trindade Motta, Karin-Soares Cunha

**Affiliations:** 1 Graduate Program in Pathology, School of Medicine, Universidade Federal Fluminense, Niterói, RJ, Brazil; 2 Neurofibromatosis National Center (Centro Nacional de Neurofibromatose), Rio de Janeiro, RJ, Brazil; 3 Department of Pathology and Oral Diagnosis, School of Dentistry, Universidade Federal do Rio de Janeiro, Rio de Janeiro, RJ, Brazil; 4 Department of Immunology and Microbiology, School of Medicine, Centro Universitário Serra dos Órgãos, Rio de Janeiro, Brazil; 5 Instituto de Puericultura e Pediatria Martagão Gesteira, School of Medicine, Universidade Federal do Rio de Janeiro, Rio de Janeiro, Brazil; 6 Department of Statistics, School of Mathematics, Universidade Federal Fluminense, Niterói, RJ, Brazil; 7 Department of Orthodontics, School of Dentistry, Universidade Federal Fluminense, Niterói, RJ, Brazil; 8 Department of Pathology, School of Medicine, Universidade Federal Fluminense, Niterói, RJ, Brazil

## Abstract

**Background:**

Oral manifestations are common in neurofibromatosis 1 (NF1), and include jaws and teeth alterations. Our aim was to investigate the craniomaxillofacial morphology of Brazilian children, adolescents and adults with NF1 using cone beam computed tomography.

**Material and Methods:**

This study was conducted with 36 Brazilian individuals with NF1 with ages ranging from 4 to 75. The participants were submitted to anamnesis, extra and intraoral exam and cephalometric analysis using cone beam computed tomography. Height of the NF1 individuals was compared to the length of jaws and skull base. The results of the cephalometric measurements of the NF1 group were compared with a control group paired by age, gender and skin color.

**Results:**

Individuals with NF1 had lower maxillary length (*p*<0.0001), lower mandibular length (*p*<0.0001), lower skull base length (*p*<0.0001). In children and adolescents, the mandible was more posteriorly positioned (*p*=0.01), when compared with the control group. There was no association between jaws and skull base length with the height of the individuals with NF1.

**Conclusions:**

Brazilian children, adolescents and adults with NF1 have short mandible, maxilla and skull base. Moreover, children and adolescents present mandibular retrusion.

** Key words:**Neurofibromatosis 1, oral manifestations, craniofacial abnormalities, cone beam computed tomography.

## Introduction

Neurofibromatosis 1 (NF1, OMIM 162200) is one of the most common genetic disorders, and presents full penetrance and variable phenotypic expression. It is caused by mutations in the *NF1* gene, located on chromosome 17q11.2. Neurofibromin, the product of this gene, is a RasGTPase activating protein that activates the intrinsic GTPase-activity of Ras and thus negatively regulates its role in signal transduction. Therefore, NF1 is considered a Rasopathy, which is a term used for a group of neurodevelopment syndromes caused by germline mutations in genes encoding proteins involved in RAS/MAPK signaling pathway (1). The main clinical features of NF1 include multiple neurofibromas, Lisch nodules, café-au-lait spots, and skin-fold freckle-like lesions (2). Bone alterations are other important manifestations of NF1 and include macrocephaly, scoliosis, osteoporosis, bowing of long bones, sphenoid wing dysplasia, and short stature (3).

Oral manifestations in individuals with NF1 occur in around 70% of all cases, and may affect the soft tissues, teeth and jaws.(4–10) The most common oral manifestations in NF1 are the enlargement of fungiform papillae of the tongue, intraoral neurofibromas, alveolar ridge deformities, enlargement of the mandibular canal and mandibular foramen, and hyposalivation (4-10). Some of these jaws alterations are associated with the presence of plexiform neurofibroma of the second and third branches of the trigeminal nerve (6,8).

Short stature and facial dysmorphology are some of the clinical features that are common to all Rasopathy syndromes (1,11). Nevertheless, little is known about the specific craniomaxillofacial alterations in NF1. There are three previous studies that evaluated the facial pattern of individuals with NF1: one was performed with Finnish patients, the other with an American and the third with a German sample (12–14). The first two studies reported the presence of short mandible, maxilla and skull base in adults when compared with a control group, but these alterations were not statistically significant in children and adolescents with NF1 (12,13). The only alteration found in the non-adult sample with NF1 in these studies was the presence of facial horizontal growth (13). Recently, Friedrich *et al.* (14) did not find significant alteration of the facial skeleton in their sample with NF1.

Since craniomaxillofacial morphology differs between ethnic groups, it becomes important to study the abnormalities in different populations to understand the impact of NF1 in this context. The aim of this study was to investigate the craniomaxillofacial morphology of Brazilian children, adolescents and adults with NF1 using cone beam computed tomography (CBCT).

## Material and Methods

This is a case control and prospective study conducted from 2011 to 2015, at Universidade Federal Fluminense (Oral Diagnostic Service of Hospital Universitário Antônio and Department of Orthodontics, School of Dentistry), and Universidade Federal do Rio de Janeiro (UFRJ; Oral Diagnosis Service and Oral Radiology Service), Brazil. The study was approved by the local Ethics Committee (#31640/2012) and all the participants’ legal sponsors gave their written informed consent to participate. The procedures followed were also in accordance with the Helsinki Declaration of 1975, as revised in 1983.

- Sample size calculation

OpenEpi online software version 3.03 (Open Source Epidemiologic Statistics for Public Health, Atlanta, USA) was used to calculate the sample size, considering a power test of 80% and *p*<0.05. For this, we used the means and standard deviations of the cranial base (SN), maxilla (ANS-PNS; SNA) and mandible (Go-Me; GoGn.SN; SNB) from an initial sample of seven Brazilian children and adolescents with NF1 as well as seven Brazilian adults with NF1, using CBCT. These measures were compared with a control group matched by gender, age and skin color, through CBCT images obtained from the Department of Orthodontics. As a result, it was possible to establish a sample of 36 individuals for each group.

- Sample

The study group was composed of 36 individuals with the diagnosis of NF1 according to the criteria of the National Institutes of Health (NIH).(2) All participants with NF1 were submitted to anamnesis, extra and intraoral exam and cephalometric analysis using CBCT. Participants who were not able to cooperate during clinical and/or radiological examination were not included in this study, as well as pregnant women and patients with facial plexiform neurofibroma. The height of the participants with NF1 was also obtained and compared to the length of jaws and skull base.

In order to compare the results of the cephalometric measurements of the individuals with NF1, we included a control group composed of 36 CBCT images from non-NF1 individuals that were digitally stored at the Department of Orthodontics and Oral Radiology Service. The control group was paired by gender, age and skin color with the study group. The skin color was included because of the variation of ethnic characteristics in the craniomaxillofacial pattern, and was classified by the researchers. For both groups, individuals with history of facial trauma, orthodontic intervention and orthognathic surgery prior to obtaining the CBCT images were excluded.

- Cone beam computed tomography acquisition

CBCT images of the study group were obtained with the volumetric scanner Kodak 9500 3D System Cone Beam (FOV: 18 cm x 21 cm; image acquisition time: 10.8 seconds; voltage: 90 kV; tube current: 10 mA; voxel size: 0.3 mm, focal spot: 0.7 mm; focus-to-detector distance: 73 cm; absorbed dose: 1,467 mGy/cm2; effective dose: 14.6 µSv/cm2). Image processing was performed with Kodak Dental Imaging System Image Capture Software (Carestream Health, Atlanta, USA), and stored in DICOM format.

The CBCT images from the control group were previously obtained with iCat-3D scanner and processed by its own software (2.0.21 Xoran Tecnologies, USA) to create a DICOM file. For both groups, patient’s head was positioned with Frankfurt’s horizontal plane parallel to the ground and the mandible was closed in maximum intercuspation. DICOM files from both groups were imported to InVivo software 5.0 (Anatomage, USA) and a single calibrated researcher performed all analyses using the 3D Analysis tool. This tool guides the operator during the identification of cephalometric landmarks, showing anatomical regions in 2D multiplanar slices or in three-dimensional reconstruction surfaces, allowing precise point marking to build a customized cephalometric analysis.

- Facial bone symmetry analysis

As usual the cephalometric analysis was performed on the left size. Therefore, significant facial bone asymmetry was used as an exclusion criterion since major bone asymmetries could have influence on the anteroposterior and vertical analysis. To investigate the bone facial symmetry, cephalometric points were selected according to a previous study (15). The points located in the midline were initially defined: point A, Nasion (N), Gnathion (Gn), Menton (Me) and Sella (S). Then, the bilateral points were selected: right Gonion (Gor), left Gonion (Gol), right condylar (Cdr), left condylar (Cdl), right condyle (Cor) and left condyle (Col).

Bilateral measurements were obtained through plans traced from the points of the right and left sides, using the central plan as a reference. This plan was generated by connecting the central points A, N, Gn, Me and S. The generated values were compared in a paired manner, differentiating the right side and the left side.

- Cephalometric analysis

Cephalometric analysis was planned using points, planes and angles from the analysis of Ricketts, Tweed, Steiner and McNamara, using the software tools in 2D and 3D images. These measures were selected to define, mainly, the size of skull base and jaws. The selected cephalometric measurements are shown in  Table 1.

**Table 1:**
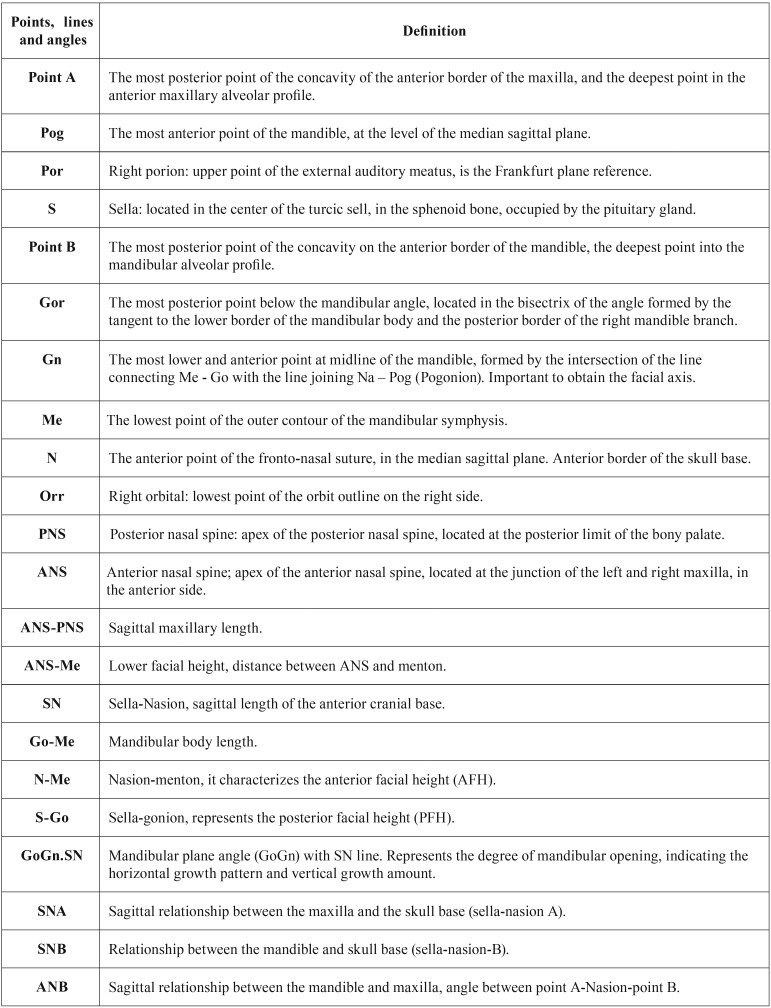
Cephalometric points, lines and angles used in the study.

Jarabak´s index, which measures the direction of facial growth, was determined through the relationship between anterior facial height (AFH) and posterior facial height (PFH).

- Statistical analysis

Excel (Microsoft Corporation, Microsoft Excel® 2010, USA) and SPSS 20.0 (IBM® SPSS®, USA) softwares were used for analysis. For the analysis of facial bone symmetry, the values of the right and left side of the individuals were compared by Student’s paired T test, to identify the presence of significant asymmetry. The cephalometric measurements were analyzed using the Student’s paired T test, comparing the study group with the control group. The linear measurements for the length of the mandible (Go-Me), maxilla (ANS-PNS) and skull base (SN) were compared with the height of the individuals with NF1, using Pearson´s regression coefficient. *P*-values ≤ 0.05 were considered significant.

## Results

According to the bilateral results, there was no facial bone asymmetry in any group; so all the individuals were included in the cephalometric analysis. The results of the evaluation of the facial bone symmetry are shown in  Table 2.

**Table 2:**
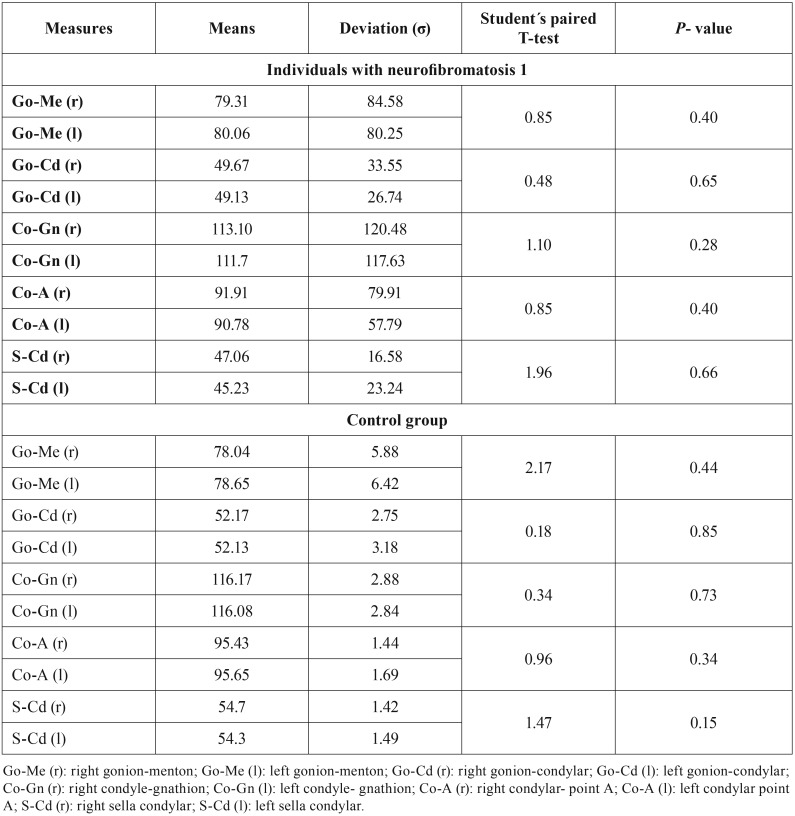
Results of the facial bone symmetry analysis.

The facial clinical aspects of the individuals with NF1 are shown in supplemental Figure 1 and 2. Of the 36 individuals included in each group (study and control group), 22 (61%) were female and 14 (39%) were male, with ages ranging from four to 75 years (mean 28 years). According to the classification by age, there were nine (25%) children (12 years of age and younger), 11 (30%) adolescents (13-18 years of age) and 16 (45%) adults (19 years of age and older).

**Figure 1:**
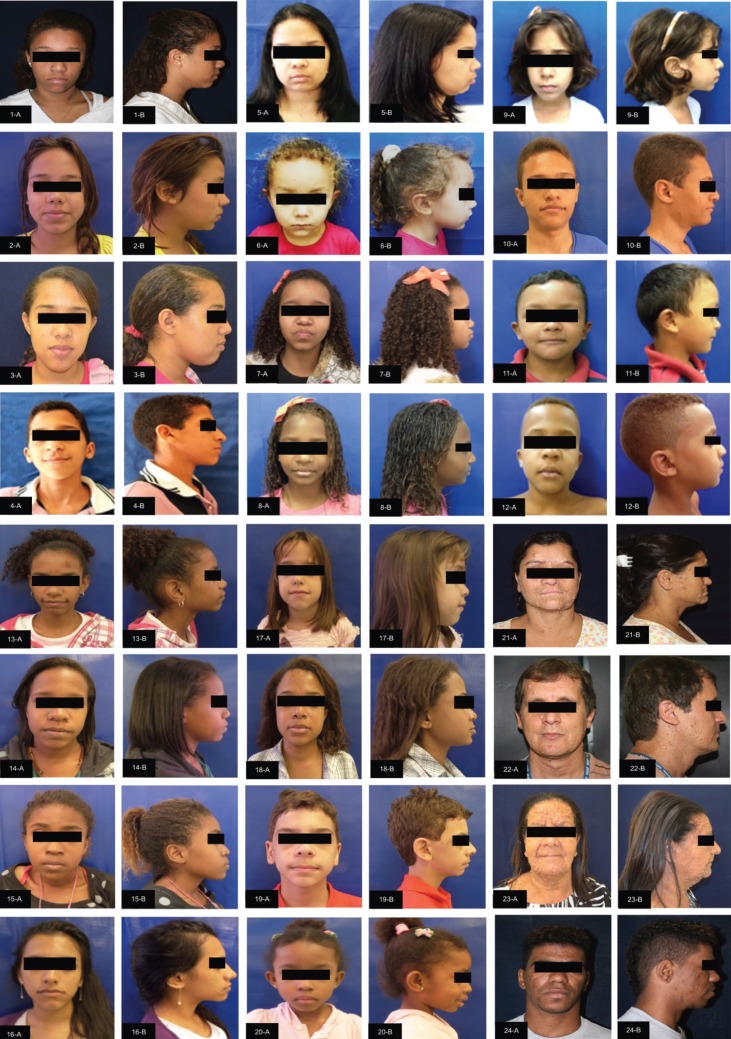
Facial clinical aspects of the individuals with neurofibromatosis 1 (patient 1-24) Front (A) and lateral (B) views.

**Figure 2:**
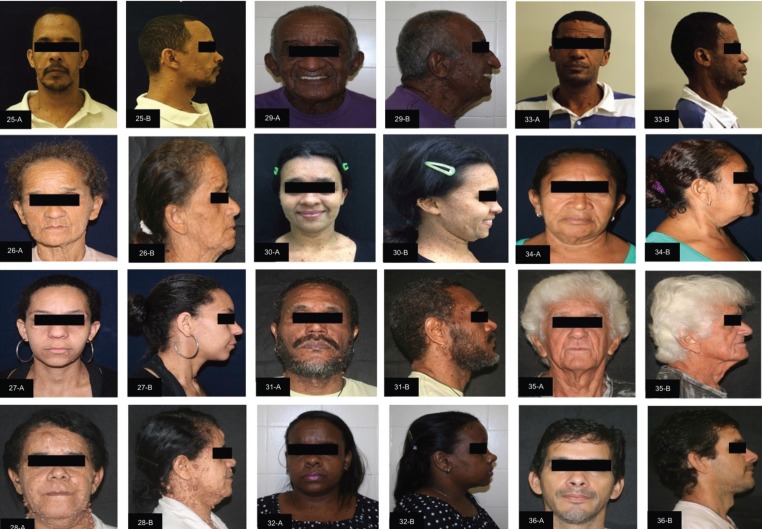
Facial clinical aspects of the individuals with neurofibromatosis 1 (patient 25-36). Front (A) and lateral (B) views.

- Comparison of the cephalometric analysis between individuals with neurofibromatosis 1 and references values

Initially, cephalometric values of individuals with NF1 were compared with the reference values (Ricketts, Tweed, Steiner and McNamara analyses). Sixteen (44%) individuals with NF1 had lower values of the SNA angle compared with the reference values, characterizing a maxillary retrusion. Reduced SNB was present in 20 (55%) participants with NF1. In the evaluation of the ANB angle, 17 (47%) individuals with NF1 had values between 0 and 4o, which is considered normal (Class I skeletal pattern). The remaining 18 individuals with NF1 (50%) showed higher values than 4o (Class II ske-letal pattern). In the linear measurement (ANS-PNS), 30 (83%) individuals with NF1 presented lower value, which characterizes short maxilla.

**Figure 3:**
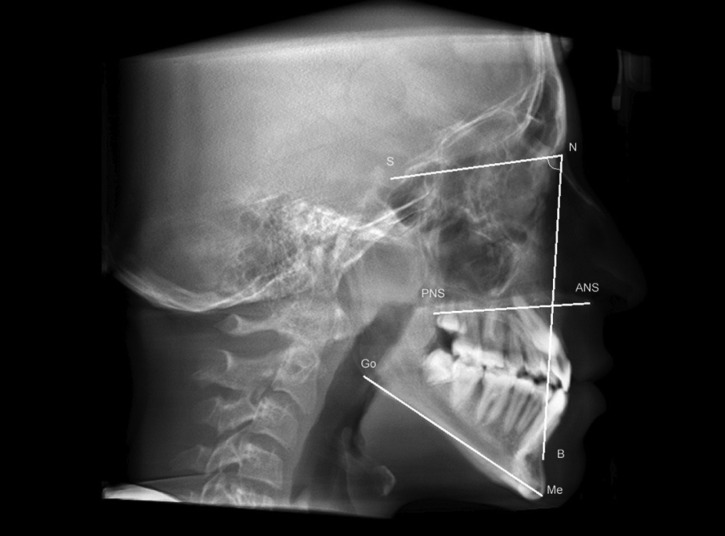
Measures that had significant differences between the NF1 and control groups. SN: sella-nasion line (skull base length); NB: nasion-B line; SNB: Relationship between the mandible and skull base (sella-nasion-B). ANS-PNS: anterior nasal spine-posterior nasal spine line (maxilla length); Go-Me: gonion-menton line (mandible length).

There were 19 (53%) individuals with NF1 with reduction of the Go-Me value, and 23 (64%) with GoGn.SN values lower than the reference, showing shorter mandibular body length and a horizontal growth pattern, respectively. The length of the skull base (SN) was reduced in 31 (86%) of the individuals with NF1. In Figure 3, we show the measures that had significant differences between the NF1 and control groups.

- Cephalometric analysis comparing individuals with neurofibromatosis 1 and control group

The results are shown in  Table 3. There was no statistically significant difference of the SNA values between individuals with NF1 and controls (paired Student´s T-test; *p*=0.26). The NF1 group had lower SNB values compared with the control group (paired Student´s T-test, *p*=0.01), showing that individuals with NF1 had mandibular retrusion. The NF1 group had normal va-lues for ANB angle, which indicate a normal horizontal relationship of the maxilla and mandible, and there was no difference comparing with the controls (paired Student´s T-test; *p*=0.09). Respecting the maxillary length (ANS-PNS), individuals with NF1 had lower measures than the controls (paired Student´s T-test; *p*<0.0001).

Individuals with NF1 had lower mandibular length values (Go-Me) than the control group (paired Student’s T-test; *p*<0.0001), which means that they had shorter mandible ( Table 3). There was no statistically significant difference between both groups for GoGn.SN va-lues (paired Student’s T-test; *p*=0.11).

Individuals with NF1 had lower values of skull base length (SN) than controls (paired Student´s T-test; *p*<0.0001). Jarabak´s analysis showed horizontal facial growth pattern (brachyfacial type) in both NF1 group (68%) and the control group (69%). There was no diffe-rence between the group with NF1 (mean 0.68; *p*=1.09) and the controls regarding the facial type (paired Student´s T-test; *p*=0.19).

When evaluating the cephalometric measures stratified by age (children/adolescents, and adults), the results were similar, except for the SNB angle, that had statistical significant differences between the group with NF1 and the control group only in children and adolescents, not in adults (Student´s T-test, *p*=0.01). The results are presented in  Table 3.

**Table 3:**
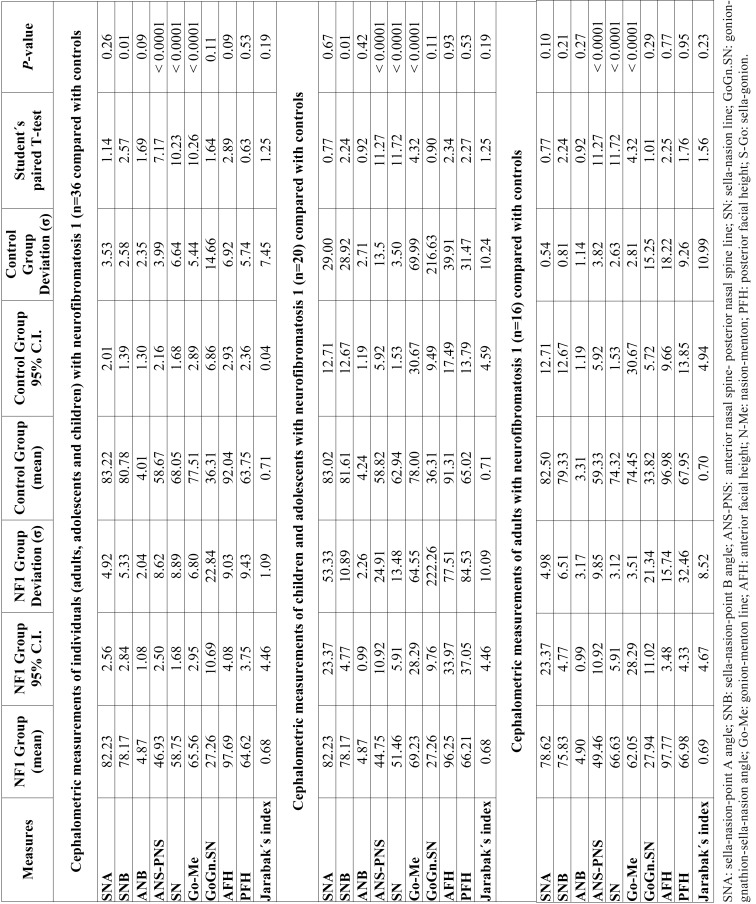
Results of the cephalometric analysis of individuals with neurofibromatosis 1 compared with controls.

- Cephalometric analysis in individuals with neurofibromatosis 1 comparing to height

 Table 4 summarizes the results. There was no association between mandibular length (Pearson´s correlation; *p*=0.478), maxillary length (Pearson´s correlation; *p*=0.700) and skull base length (Pearson´s correlation; *p*=0.570) with the height of the individuals with NF1. Analyzing the correlation by sex, there was also no association between the individual’s height with mandibular length (Pearson´s correlation; male: *p*=0.672 / female: *p*=0.087), maxillary length (Pearson´s correlation; male: *p*=0.202 / female: *p*=0.539) and skull base length (Pearson´s correlation; male: *p*=0.944/ female: *p*=0.570).

**Table 4:**
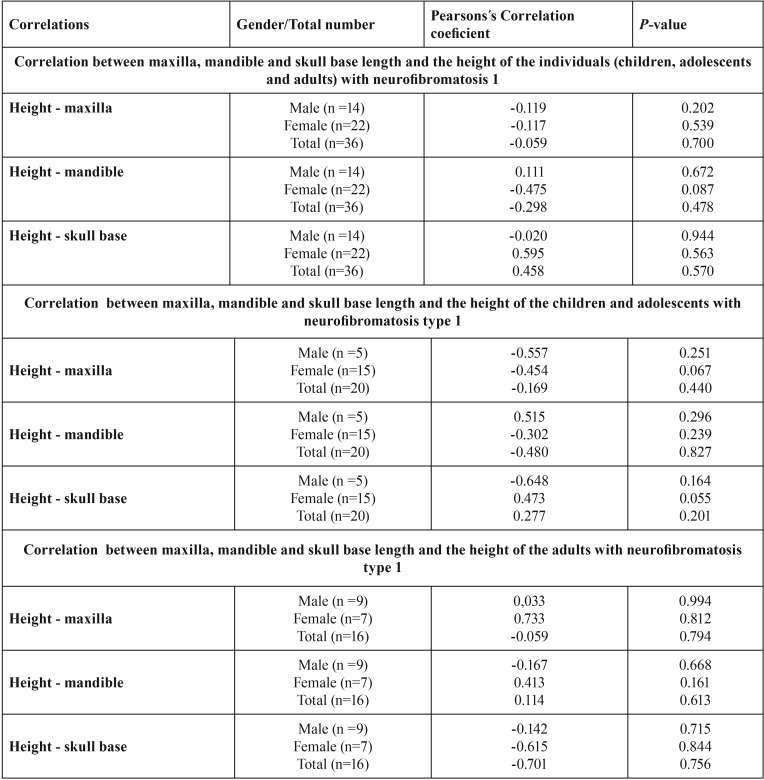
Correlation between maxilla, mandible and skull base length and the height of the individuals with neurofibromatosis type 1.

## Discussion

The use of CBCT images for cephalometric analyses in this study was based on their benefits over conventional radiographies (16-19). It was possible to obtain images with actual size, avoiding superposition of structures, thus presenting better accuracy in marking the anthropometric points, and ensuring greater reliability of the identification of anatomic structures (16-19). CBCT also permits 3D reconstructions and emulation of 2D conventional projections (16-19). Although computed tomographies (CT) require higher doses than conventional radiographies, CBCT exposes patients to lower doses of radiation comparing with conventional CT (20). For example, head CT requires doses between 995 and 1.160 µSv, whereas the large FOV CBCT requires 93 to 260 µSv for the Kodak 9500 3D, which was the equipment used in this study (19). Even using the large FOV, it was possible to obtain lower radiation doses with CBCT comparing with a complete oral radiographic exam (periapical, bite-wing, panoramic and lateral radiographies).(19) Moreover, the radiation dose of CBCT or complete oral radiographic exam is much lower than other conventional radiographies, i.e. spine, abdomen or pelvis (1.5-8 mSv) (20).

Although CBCT presents important advantages over conventional radiographies, there are no previous studies that investigated the craniofacial alterations in NF1 using this technique. The use of CBCT images in the present study allowed a previous accurate facial symmetry analysis as an exclusion criterion, avoiding the influence of bone asymmetry on anteroposterior and vertical measurements, thus resulting in a more homogeneous sample. Based on CBTC images, we show in this study that Brazilian individuals with NF1 have short jaws and skull base, as well as mandibular retrusion.

Short jaws and skull base were first shown in individuals with NF1 in a study with a Finnish sample, performed by Heervä *et al.* (12), based on conventional radiographies. Nevertheless, statistical differences comparing with a control group were present only in adults (n=51) and not in children/adolescents (n=30). According to the authors, it probably happened because of the small number of participants in the latter group. Similarly, also using conventional radiographies, the study of Cung *et al.* (13) conducted with an American sample, found short mandible, maxilla and skull base only in the adult sample (n=78), but not in children and adolescents with NF1 (n=23), compared with controls.

Although our total sample was smaller than other two previous studies (12,13), our sample size of children and adolescents was similar to the study of Cung *et al.* (13), and we found a significant statistical difference in the values of mandibular, maxilla and skull base length comparing the NF1 group with the controls not only in adults but also in children and adolescents. In the facial growth analysis, Heervä *et al.* (12) showed significant difference between adolescents with NF1 and controls, demonstrating a short growth pattern. It is important to emphasize that the study of Heervä *et al.* (12) was conducted with Finnish individuals, which is an ethnic group with Caucasian features, and the study of Cung *et al.* (13) was performed with American whites with NF1. Our sample is different because it was composed predominantly of individuals with a shorter face, which is the result of miscegenation of the Brazilian population. Although the general Brazilian population has shorter face comparing with other ethnic groups, we show in our study that Brazilian individuals with NF1 have even shorter jaws and skull base comparing with the controls.

Different from our results where we found significant mandibular retrusion (reduced SNB) in children and adolescents, Heervä *et al.* (12) and Cung *et al.* (13) did not find statistically difference comparing with the controls. Significant maxillary retrognathism (reduced SNA) was reported in the study of Heervä *et al.* (12), but not in the one performed by Cung *et al.* (13). In our sample, although SNA reduction was present in 44% of the individuals with NF1, it was not statistically significant comparing with the controls. Maybe with a larger sample, we could find statistical differences of the SNA values between the individuals with NF1 and the control group.

Friedrich *et al.* (14) recently performed a cephalometric study with a larger sample of individuals with NF1 (n=172), with ages between 4 and 78 years. In their study, although there was some shortening in the measurements of the skull and jaws in patients with NF1 without facial plexiform neurofibromas (n=96), when the results were compared with the control group, there were no statistical significance differences (14). Nevertheless, the control group of their study was composed of only 29 individuals with NF1, from ages between 16 to 35 years (14). It is important to emphasize that cephalometric values vary between individuals with different gender and age, as well as between ethnic groups. In the other two previous studies on cephalometric analysis of NF1 individuals (12,13), as well as in ours, short jaws and skull base were present in the NF1 population and there were significant statistical differences when compared with the controls that were matched by age and sex. In our study, since Brazilian population is miscegenated, the control group was also matched by skin color.

The previous study with Finnish individuals with NF1 found correlation between stature and short jaws in children and adolescents, as well as with short skull base, demonstrating that short children with NF1 have short craniomaxilofacial bones (12). These results were not present in adulthood, and the authors suggested that there might be some alterations in growth during puberty (12). Interestingly, Cung *et al.* (13) found, in the adult population, but not in children, correlation between stature and some cephalometric changes (facial height, and length of maxilla), but other the alterations (e.g. ANB) were independent of height. In our sample, there was no statistically significant correlation between cephalometric measures and height of children and adolescents with NF1, demonstrating that the presence of short jaws and skull base in our sample were independent of the individuals’ stature.

Neurofibromin is expressed in osteoblasts, osteocytes, osteoclasts, and chondrocytes (21), and previous in vitro and in vivo studies have shown that neurofibromin is necessary for proper endochondral ossification and osteoblast differentiation (22,23). Mandible, maxilla and some bones of the skull base (sphenoid body, occipital base) develop from endochondral ossification. Moreover, intramembranous ossification also occurs in the maxilla and mandible, as well as in sphenoid wings and occipital squama. The results of our study together with the two previous ones that showed similar craniomaxillofacial morphology alterations in individuals with NF1 (12,13) suggest that neurofibromin is important for the correct anterior-posterior growth of skull base, maxilla and mandible. Interestingly, mice lacking neurofibromin in osteochondroprogenitor cells exhibited alterations in bones derived from endochondral ossification, including shortening of skull base, and maxilla (23), as seen in our sample and in the other two studies with humans (12,13). Moreover, mice also presented shortening of the zygomatic and squamosal bones, associated with teeth malocclusion, whereas craniofacial bones derived from intramembranous ossification appeared normal (23).

Future studies for a better understanding of the consequences of the craniomaxillofacial morphology alterations in NF1 are important since it is known from the general population that facial bone alterations can influence the masticatory and facial muscle activity, and can affect physiological functions, such as speech, chewing and swallowing (24-26). Interestingly, Class III malocclusion and posterior unilateral cross bite are highly prevalent in children with NF1 compared with controls (10,27). The correlation between these occlusal abnormalities and the craniofacial malformations in individuals with NF1 should be further investigated.

In conclusion, we show in this study with a Brazilian sample that children, adolescents and adults with NF1 have short mandible, maxilla and skull base. Moreover, children and adolescents present mandibular retrusion. Our results suggest that NF1 affects the craniofacial morphology.

## References

[1] Cizmarova M, Kostalova L, Pribilincova Z, Lasabova Z, Hlavata A, Kovacs L (2013). Rasopathies - dysmorphic syndromes with short stature and risk of malignancy. Endocr Regul.

[2] Neurofibromatosis (1988). Conference statement. National Institutes of Health Consensus Development Conference. Arch Neurol.

[3] Elefteriou F, Kolanczyk M, Schindeler A, Viskochil DH, Hock JM, Schorry EK (2009). Skeletal abnormalities in neurofibromatosis type 1: approaches to therapeutic options. Am J Med Genet. A.

[4] Shapiro SD, Abramovitch K, Van Dis ML, Skoczylas LJ, Langlais RP, Jorgenson RJ (1984). Neurofibromatosis: oral and radiographic manifestations. Oral Surg Oral Med Oral Pathol.

[5] D'Ambrosio JA, Langlais RP, Young RS (1988). Jaw and skull changes in neurofibromatosis. Oral Surg Oral Med Oral Pathol.

[6] Friedrich RE, Giese M, Schmelzle R, Mautner VF, Scheuer HA (2003). Jaw malformations plus displacement and numerical aberrations of teeth in neurofibromatosis type 1: a descriptive analysis of 48 patients based on panoramic radiographs and oral findings. J Craniomaxilofac Surg.

[7] Cunha KSG, Barboza EP, Dias EP, Oliveira FM (2004). Neurofibromatosis type I with periodontal manifestation. A case report and literature review. Br Dent J.

[8] Cunha KS, Rozza-de-Menezes RE, Andrade RM, Almeida L, Janini M, Geller M (2015). Oral manifestations of neurofibromatosis type 1 in children with facial plexiform neurofibroma: report of three cases. J Clin Pediatr Dent.

[9] Cunha K, Rozza-de-Menezes R, Luna E, Almeida L, Pontes R, Almeida P (2015). High prevalence of hyposalivation in individuals with neurofibromatosis 1: a case-control study. Orphanet J Rare Dis.

[10] Bardellini E, Amadori F, Flocchini P, Conti G, Piana G, Majorana A (2011). Oral findings in 50 children with neurofibromatosis type 1. A case control study. Eur Acad Paediatr Dent.

[11] Hernández-Martín A, Torrelo A (2011). Rasopathies: developmental disorders that predispose to cancer and skin manifestations. Actas Dermo-Sifiliográficas.

[12] Heervä E, Peltonen S, Pirttiniemi P, Happonen RP, Visnapuu V, Peltonen J (2011). Short mandible, maxilla and cranial base are common in patients with neurofibromatosis 1. Eur J Oral Sci.

[13] Cung W, Freedman LA, Khan NE, Romberg E, Gardner PJ, Bassim CW (2015). Cephalometry in adults and children with neurofibromatosis type 1: Implications for the pathogenesis of sphenoid wing dysplasia and the "NF1 facies. " Eur J Med Genet.

[14] Friedrich RE, Lehmann JM, Rother J, Christ G, Zu Eulenburg C, Scheuer HT (2017). A lateral cephalometry study of patients with neurofibromatosis type 1. J craniomaxillofac Surg.

[15] Rothier EKC (2011). Comparação da simetria craniana através de imagens obtidas de tomografia computadorizada cone beam (Thesis). Universidade Federal Fluminense.

[16] Cevidanes LH, Styner MA, Proffit WR (2006). Image analysis and superimposition of 3-dimensional cone-beam computed tomography models. Am J Orthod Dentofacial Orthop.

[17] Hassan B, van der Stelt P, Sanderink G (2009). Accuracy of three-dimensional measurements obtained from cone beam computed tomography surface-rendered images for cephalometric analysis: influence of patient scanning position. Eur J Orthod.

[18] Grauer D, Cevidanes LS, Styner MA, Heulfe I, Harmon ET, Zhu H (2010). Accuracy and landmark error calculation using cone-beam computed tomography-generated cephalograms. Angle Orthod.

[19] Lorenzoni DC, Bolognese AM, Garib DG, Guedes FR, Sant'Anna EF (2012). Cone-beam computed tomography and radiographs in dentistry: aspects related to radiation dose. Int J Dent.

[20] Scarfe WC, Farman AG, Sukovic P (2006). Clinical applications of cone-beam computed tomography in dental practice. J Can Dent Assoc.

[21] Kuorilehto T, Nissinen M, Koivunen J, Benson MD, Peltonen J (2004). NF1 tumor suppressor protein and mRNA in skeletal tissues of developing and adult normal mouse and NF1-deficient embryos. J Bone Miner Res.

[22] Kolanczyk M, Kossler N, Kühnisch J, Lavitas L, Stricker S, Wilkening U (2007). Multiple roles for neurofibromin in skeletal development and growth. Hum Mol Genet.

[23] Wang W, Nyman JS, Ono K, Stevenson DA, Yang X, Elefteriou F (2011). Mice lacking Nf1 in osteochondroprogenitor cells display skeletal dysplasia similar to patients with neurofibromatosis type I. Hum Mol Genet.

[24] Lowe AA (1980). Correlations between orofacial muscle activity and craniofacial morphology in a sample of control and anterior open-bite subjects. Am J Orthod.

[25] Kjellberg H, Beiring M, Albertsson Wikland K (2000). Craniofacial morphology, dental occlusion, tooth eruption, and dental maturity in boys of short stature with or without growth hormone deficiency. Eur J Oral Sci.

[26] Tsai HH (2003). Dental crowding in primary dentition and its relationship to arch and crown dimensions. J Dent Child (Chic).

[27] Bardellini E, Tonni I, Micheli R, Molinaro A, Amadori F, Flocchini P (2016). Occlusal traits in children with neurofibromatosis type 1. Orthod Craniofac Res.

